# Global constraint principle for microbial growth laws

**DOI:** 10.1073/pnas.2515031122

**Published:** 2025-10-03

**Authors:** Jumpei F. Yamagishi, Tetsuhiro S. Hatakeyama

**Affiliations:** ^a^Center for Biosystems Dynamics Research, RIKEN, Kobe 650-0047, Japan; ^b^Universal Biology Institute, Graduate School of Science, The University of Tokyo, Tokyo 113-0033, Japan; ^c^Earth-Life Science Institute, Institute of Future Science, Institute of Science Tokyo, Tokyo 152-8550, Japan

**Keywords:** cellular growth, metabolism, resource allocation, convex optimization, dual problem

## Abstract

Understanding complex living systems requires identifying universal phenomenological laws that are independent of species and molecular-biological details. The Monod equation is a cornerstone of phenomenological microbial growth laws, depicting the growth rate as a saturating function of a single substrate. Because of its similarity in functional form to the Michaelis–Menten equation, cellular growth is often thought to be limited by a single reaction. However, cellular growth generically results from the coordination of thousands of metabolic reactions and diverse intracellular limited resources. Consequently, the mechanistic origins of the Monod equation remain controversial, and its extension has encountered limitations. Here, we propose the global constraint principle for cellular growth: As one nutrient becomes more available, other intracellular resources become limiting, driving transitions to distinct modes of resource allocation. Based on a general framework of constraint-based modeling and its dual formulations, we mathematically prove that, in general, microbial growth kinetics curves are monotonically increasing and concave with respect to nutrient availability. Numerical simulations using genome-scale *Escherichia coli* models with proteome allocation, molecular crowding, and membrane capacity constraints reproduce these features in a multiphasic manner. In contrast to the original Monod’s growth law, the global constraint principle also captures the dependence of microbial growth on the availability of multiple nutrients, by generalizing the Liebig’s law of the minimum, another phenomenological growth law for higher organisms, into a terraced landscape of diminishing returns. It thus integrates the classical phenomenological laws proposed by Monod and Liebig into a comprehensive theory of cellular growth.

## Significance

Cellular growth kinetics is a classical and fundamental problem in biology, traditionally described by Monod’s law. However, its universality has long remained uncertain. While the universal validity of this law itself is not argued, we prove that its key features—monotonicity and concavity in nutrient dependence—are universal consequences of intracellular resource allocation, providing a systematic theoretical basis for cellular growth kinetics. Unlike Monod’s model, which implies a local view centered on a single limiting reaction, our framework highlights global constraints on growth, thus generalizing the classical idea of Liebig’s law into a modern theory. By unifying these classical phenomenological laws of Monod and Liebig, our theory paves the way for a more comprehensive understanding of organismal growth.

To understand complex living systems, universal phenomenological laws independent of specific species or molecules are of paramount importance (1). In particular, the dependence of cellular growth rates on environmental conditions (and cellular physiological states) has been an important problem in biology for over a century (2–5). The classical phenomenology, originally proposed by Monod in the 1940s (2), states that microbial growth kinetics, namely the dependence of the specific growth rate μ on substrate availability, generally follows the Monod equation:

[1] $$\begin{array}{c} \mu \left(\left[S\right]\right)=\mu_{\max}\frac{\left[S\right]}{K_{S}+\left[S\right]}, \end{array}$$

where $\mu_{\max}$ is the maximum growth rate and [S] is the environmental concentration of the growth-limiting substrate S with its half-saturation concentration $K_{S}$.

**Fig. 1.**
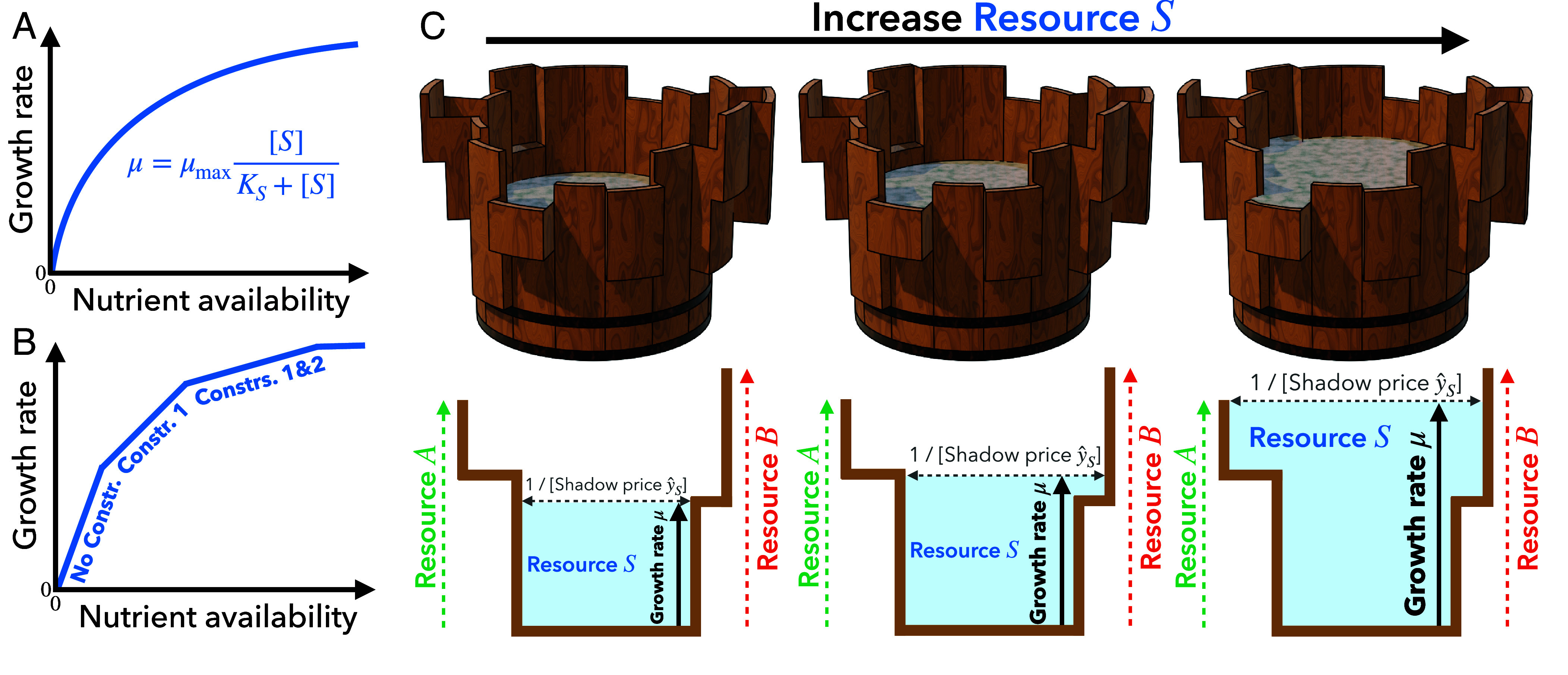
Microbial growth kinetics. (*A*) Monod’s microbial growth model. (*B*) Global constraint principle for the microbial growth kinetics curve. Cell growth is globally limited by constraints on intracellular resource allocation. When the availability of the focal nutrient is scarce, no other constraints limit growth (“No Constr.”). As nutrient availability increases, some global constraint (“Constr. 1”) becomes effective and reduces the growth return from the focal nutrient. With further increases, another global constraint (“Constr. 2”) also becomes growth-limiting. In this way, the sequential activation of global constraints results in diminishing returns in cellular growth. (*C*) Terraced Liebig’s barrel (*Top*) and its sectional view for the case with a focal substrate S and the other resources A and B (*Bottom*). As S is poured into the barrel, the surface height corresponds to the growth rate μ, where the height of each stave represents the availability of each resource other than S. The water surface area in the barrel is therefore $\partial I_{S}/\partial \mu$, which coincides with the inverse of the shadow price ${\hat{y}}_{S}\;=\;\partial \mu /\partial I_{S}$ (i.e., the slope of the growth kinetics curve). Each stave spreads out in a stepwise manner, reflecting the metabolic shifts to pathways with lower growth yield from S due to additional global constraints.

The mechanistic basis for how the substrate limits growth is typically explained by a local biochemical bottleneck. Since the Monod equation has the same functional form as the Michaelis–Menten equation, a single biochemical process is usually assumed to locally limit cell growth (6–9) (Fig. 1*A*). In previous hypotheses, this single “black box” could be the substrate transport into cells (6, 10), the net flux of respiration (11), or the (coarse-grained) reaction that couples catabolism and anabolism (12).

However, microbial growth is achieved collectively by the interplay of thousands of biochemical processes. There is considerable experimental evidence that cell growth is not limited by a single biochemical process alone (13, 14): For example, experimental changes in the environmental conditions of nitrogen sources can alter the dependence of growth rate on the availability of carbon sources (15, 16); and cells exhibit qualitatively different phenotypes with respect to the metabolism of nutrients for cell growth (17).

In addition, the accumulation of experimental data has shown that the experimentally observed microbial growth kinetics are not accurately captured by the Monod equation (5, 6, 18, 19). That is, the shape of actual microbial growth kinetics curves varies across species and environments and there is no longer quantitative evidence to support the exact functional form of Eq. **1**. Nevertheless, certain characteristics still appear to be universal (6, 18, 19): i) growth rate is a monotonically increasing function of nutrient availability and ii) growth rate exhibits concavity with respect to nutrient availability, in other words, there are diminishing returns to getting more and more of a nutrient.

Therefore, a novel macroscopic framework is essential to elucidate the general mechanism underlying these fundamental characteristics (i–ii) of microbial growth. We here propose the global constraint principle for the law of diminishing returns in cellular growth: as the availability of a specific nutrient increases, some other resources are gradually depleted and act as additional growth-limiting factors (Fig. 1*B*). Assuming the optimality of evolved metabolic systems, we analytically prove that global constraints on intracellular resource allocation cause the cellular growth rate to plateau with increasing nutrient availability. This framework can be seen as a generalization of Liebig’s law of the minimum, a classical phenomenological theory of plant growth, which states that the availability of multiple resources collectively limits the growth of organisms (3, 16, 20) (Fig. 1*C*). It also provides insights into the dependence of microbial growth rate on the environmental availability of multiple nutrient sources, which cannot be captured by Monod’s classical phenomenology.

## Global Constraint Principle of Cell Growth

Cell growth requires the production of biomass through metabolism. We therefore focus on how the intracellular allocation of diverse resources to metabolic processes is globally constrained by stoichiometry and the law of matter conservation. This concept can be naturally formulated within the optimization frameworks in systems biology.

Let us denote all the intracellular metabolites and metabolic reactions as M and R, respectively. Here, by decomposing each reversible reaction into two irreversible reactions (i.e., its forward and backward components), a nonnegative vector $v\;:=\;{\left\{v_{i}\right\}}_{i\in R}$ represents the fluxes of all |R| reactions. Among |M| metabolites, the set of exchangeable metabolites is denoted by E(⊂M).

In the context of systems biology, the metabolic systems of cells are assumed to maximize their growth rate, or the biomass synthesis rate, as a natural consequence of evolution (17, 21–23). Since we are considering continuously growing microbes, stationarity of intracellular concentrations of nonexchangeable metabolites m(∈M\E) is also assumed. Then, the metabolic regulation of reaction fluxes v can generally be formulated as a linear programming (LP) problem, known as constraint-based modeling (CBM) of metabolism (17, 24, 25):

[2] $$\begin{array}{cc} \underset{v\geq 0}{\text{maximize}}\;\;v_{\text{gr}}\;\;\text{s.t.} & \sum_{i\in R}S_{\mathrm{mi}}v_{i}=0\;\;\left(m\in M\backslash E\right), \end{array}$$

[3] $$\begin{array}{cc} & \sum_{i\in R}S_{\mathrm{mi}}v_{i}+I_{m}\geq 0\;\;\left(m\in E\right), \end{array}$$

[4] $$\begin{array}{cc} & \sum_{i\in R}C_{\mathrm{ai}}v_{i}\leq I_{a}\;\;\left(a\in C\right), \end{array}$$

where gr(∈R) denotes the growth or biomass synthesis reaction. $S_{\mathrm{mi}}$ is the stoichiometric coefficient representing that metabolic reaction i(∈R) produces (consumes) $|S_{\mathrm{mi}}|$ molecules of metabolite m(∈M) if $S_{\mathrm{mi}}\;>\;0$ (if $S_{\mathrm{mi}}\;<\;0$). $\sum_{i}S_{\mathrm{mi}}v_{i}$ is then the excess production of metabolite m. Thus, Eq. **2** represents that the production and degradation of internal metabolites must be balanced, and Eq. **3** represents that exchangeable metabolites m with maximal influx $I_{m}\;>\;0$ (efflux $I_{m}\;<\;0$) are taken up (degraded). The other constraints on the allocation of the nonnutrient resources C are incorporated as Eq. **4**: $I_{a}$ represents the total capacity of nonnutrient resource a(∈C), such as the total amount of enzymes (26, 27) or membrane surface area (28) or the upper bound on the Gibbs energy dissipation (29). Therefore, the LP problem (Eqs. **2**–**4**) includes standard flux balance analysis (17, 23) and its variants (27, 29–32) as well as resource balance analysis (33) as special cases. Table 1 collects all the notation in one place.

**Table 1.** Notations in the main text

Symbol	Description
μ	Growth rate ($={\hat{v}}_{\text{gr}}$)
[S]	Environmental concentration of (growth-limiting) nutrient S
M,E	Set of metabolites / exchangeable metabolites (E⊂M)
C	Set of nonnutrient resources
R	Set of reactions
gr	Growth or biomass synthesis reaction (gr∈R)
S,C	Stoichiometry matrix ($S:={\left\{S_{\mathrm{mi}}\right\}}_{m\in M,i\in R}$) and resource allocation matrix ($C:={\left\{C_{\mathrm{ai}}\right\}}_{a\in C,i\in R}$)
$I_{a}$	Maximal intake of exchangeable metabolite a(∈E) or total capacity for nonnutrient resource a(∈C)
I	Availability of the resources ($I:={\left\{I_{a}\right\}}_{a\in E\cup C}$)
$\tilde{I}$	Availability of the resources other than nutrient S ($\tilde{I}:={\left\{I_{a}\right\}}_{a\in E\cup C\backslash \{S\}}$)
$v_{i}$	Nonnegative flux of reaction i(∈R)
$v,\hat{v}$	Reaction fluxes ($v\;:=\;{\left\{v_{i}\right\}}_{i\in R}$) and its optimized solution ($\hat{v}\;:=\;{\left\{{\hat{v}}_{i}\right\}}_{i\in R}$)
${\hat{y}}_{a}$	Shadow price of metabolite a(∈M) or nonnutrient resource a(∈C)
$y,\hat{y}$	Variables of dual problem (5) ($y\;:=\;{\left\{y_{a}\right\}}_{a\in M\cup C}$) and its optimized solution (i.e., shadow prices $\hat{y}\;:=\;{\left\{y_{a}\right\}}_{a\in M\cup C}$)

The solution to the LP problem is denoted as $\hat{v}\left(I\right)$; since the optimal objective function ${\hat{v}}_{\text{gr}}\left(I\right)$ is the predicted value of the growth rate μ(I), we will hereafter refer to ${\hat{v}}_{\text{gr}}\left(I\right)$ as μ(I). Numerical calculations of the CBM method successfully explain and predict experimental data, at least for model organisms such as *Escherichia coli* strains (17, 21, 22, 34).

## Results

### Monotonicity and Concavity of Growth Kinetics Curve.

The calculated growth rate satisfies fundamental characteristics (i–ii) of the microbial growth kinetics curve. Here, we show that the dependence of the growth rate μ on the maximal influx $I_{S}$ of a given nutrient S(∈E) is generally a monotonically increasing and concave function. While such properties have sometimes been recognized empirically for specific metabolic models and parameters (35, 36), its generality can be proven mathematically as follows.

First, the monotonic increase in the growth kinetics curve $\mu (I_{S};\;\tilde{I})$, where $\tilde{I}\;:=\;{\left\{I_{a}\right\}}_{a\in E\cup C\backslash \{S\}}$ denotes the availability of the resources other than the nutrient S, is evident. When the maximal nutrient influx increases from $I_{S}$ to $I_{S}\;+\;\Delta I_{S}$ ($\Delta I_{S}\;>\;0$), any v satisfying Eq. **3** also satisfies $\sum_{i}S_{\mathrm{Si}}v_{i}\;+\;I_{S}\;+\;\Delta I_{S}\;\geq \;0$. That is, any originally feasible solution v that satisfies Eqs. **2**–**4** remains a feasible solution even after increasing nutrient availability, and thus increasing $I_{S}$ monotonically expands the feasible solution space. The growth rate $\mu \left(I_{S};\;\tilde{I}\right)\;:=\;{\hat{v}}_{\text{gr}}\left(I\right)$ is therefore a monotonically increasing function of $I_{S}$.

To prove the concavity of the growth kinetics curve $\mu (I_{S};\;\tilde{I})$, it suffices to show that its slope, $\partial \mu /\partial I_{S}$, monotonically decreases as $I_{S}$ increases. The slope coincides with the so-called shadow price ${\hat{y}}_{S}$ of nutrient S (37–39), where $\hat{y}$ denotes the solution to the dual problem defined below and its element ${\hat{y}}_{a}$ quantifies the growth return from the additional consumption of resource a(∈E∪C). In the following, we show that shadow price ${\hat{y}}_{S}\left(I_{S};\;\tilde{I}\right)$ diminishes monotonically with $I_{S}$.

The dual problem to the primal LP problem (Eqs. **2**–**4**) is given as (24, 37, 40) (see also *SI Appendix*, Text S1 for the derivation and a simple example):

[5] $$\begin{array}{cc} \underset{y\in R^{M\cup C}}{\text{minimize}}\sum_{a\in E\cup C}I_{a}y_{a}\;\;\text{s.t.} & \left(\begin{array}{cc} -S^{⊤} & C^{⊤} \end{array}\right)y\geq 1_{\text{gr}},\\ & y_{a}\geq 0\;\;\left(a\in E\cup C\right). \end{array}$$

Here, $1_{\text{gr}}$ denotes a |R|-dimensional vector, where the gr-th element is one and all other elements are zero. From the duality theorem (40), the maximized objective function of the primal problem is equal to the minimized objective function of the dual problem (Eq. **5**): i.e., $\mu \left(I\right)\;=\;\sum_{a}I_{a}{\hat{y}}_{a}\left(I\right)$. The optimal solution $\hat{y}$ to the dual problem thus satisfies ${\hat{y}}_{a}\;=\;\partial \mu /\partial I_{a}$ for arbitrary a(∈E∪C). To prove the monotonicity of ${\hat{y}}_{S}\left(I_{S};\;\tilde{I}\right)$, let us define a vector $I^{\prime }$ which differs from the vector I only in the S-th element: i.e., $I_{S}^{\prime }\;=\;I_{S}\;+\;\Delta I_{S}$ and $I_{m}^{\prime }\;=\;I_{m}$ for m(≠S). From the definition, the solutions of the minimization problem (Eq. **5**) with the parameters I and $I^{\prime }$, $\hat{y}\left(I\right)$ and $\hat{y}\left(I^{\prime }\right)$, satisfy $I\cdot \hat{y}\left(I^{\prime }\right)\geq I\cdot \hat{y}\left(I\right)$ and $I^{\prime }\cdot \hat{y}\left(I\right)\geq I^{\prime }\cdot \hat{y}\left(I^{\prime }\right)$. It follows

$$\begin{array}{cc} & I\cdot \hat{y}\left(I^{\prime }\right)+I^{\prime }\cdot \hat{y}\left(I\right)\geq I\cdot \hat{y}\left(I\right)+I^{\prime }\cdot \hat{y}\left(I^{\prime }\right)\\ & \Leftrightarrow \left(I^{\prime }-I\right)\cdot \left[\hat{y}\left(I^{\prime }\right)-\hat{y}\left(I\right)\right]\leq 0\\ & \Leftrightarrow \Delta I_{S}\left[{\hat{y}}_{S}\left(I^{\prime }\right)-{\hat{y}}_{S}\left(I\right)\right]\leq 0\\ & \Leftrightarrow \frac{{\hat{y}}_{S}\left(I_{S}+\Delta I_{S};\;\tilde{I}\right)-{\hat{y}}_{S}\left(I_{S};\;\tilde{I}\right)}{\Delta I_{S}}\leq 0. \end{array}$$

The duality also allows us to prove the concavity of μ(I) as a multivariable function of I (*SI Appendix*, Text S2).

Using the shadow price, the growth kinetics curve can be formally written as

$$\begin{array}{c} \mu \left(I_{S};\;\tilde{I}\right)=\int_{I_{S,\min}}^{I_{S}}{\hat{y}}_{S}\left(I_{S};\;\tilde{I}\right)\text{d}I_{S}, \end{array}$$

which emphasizes that the curve is shaped by the cumulative marginal growth return from nutrient uptake. Notably, there exists a minimal nutrient influx $I_{S,\min}\geq \;0$ below which no feasible solutions with nonnegative growth rate (i.e., viable states) exist. If non-growth-associated maintenance energy requirements are explicitly taken into account (41), $I_{S,\min}>\;0$ becomes a finite threshold. This does not contradict the mathematical proof above, which only applies to conditions with feasible solutions, and is consistent with experimentally observed minimum substrate concentrations ${\left[S\right]}_{\min}$ required for growth (5, 14, 42). Above $I_{S,\min}$, $\mu (I_{S})$ generally represents a multiphasic, piecewise linear function (see also *SI Appendix*, Text S1 for examples).

It is thus concluded that the monotonicity and concavity of the microbial growth kinetics curve, fundamental characteristics (i-ii), are universal for LP problems including CBM. Moreover, our proof of monotonicity and concavity is generalizable to even nonlinear convex optimization problem formulations (43) (*SI Appendix*, Text S2); this generalization allows for the consideration of additional factors of cellular metabolism and growth, such as the inclusion of metabolite concentrations as variables and their relationship to reaction fluxes as constraints, which are not included in standard CBM frameworks.

Note that the growth kinetics curve discussed in the context of Monod’s law is μ([S]): Its argument is not maximal nutrient influx $I_{S}$ but nutrient concentration [S]. However, as long as the intake function $I_{S}\left(\left[S\right]\right)$ satisfies the monotonicity and concavity as in the Michaelis–Menten equation $I_{S}\;\propto \;\left[S\right]/\left(K_{\text{M}}\;+\;\left[S\right]\right)$ with the Michaelis constant $K_{\text{M}}$ or a linear transport $I_{S}\;\propto \;\left[S\right]$, the monotonicity and concavity of the growth kinetics curve, the composite function $\mu \left(\left[S\right]\right)\;=\;\mu \left(I_{S};\;\tilde{I}\right)\;\circ \;I_{S}\left(\left[S\right]\right)$, are satisfied.^*^ Notably, if the shadow price ${\hat{y}}_{S}\left(I_{S}\right)$ remains constant so that $\mu \left(I_{S}\right)\propto \;I_{S}$, then Michaelis–Menten uptake kinetics lead to the Monod equation (Eq. **1** with $K_{S}=\;K_{\text{M}}$); in contrast, if intracellular resource allocation induces diminishing returns in $\mu (I_{S})$ (i.e., ${\hat{y}}_{S}\left(I_{S}\right)$ decreases with $I_{S}$), as suggested by experimental observations (29, 44, 45), then the composite function μ([S]) will necessarily deviate from the Monod equation, even if $I_{S}\left(\left[S\right]\right)$ follows Michaelis–Menten kinetics.

**Fig. 2.**
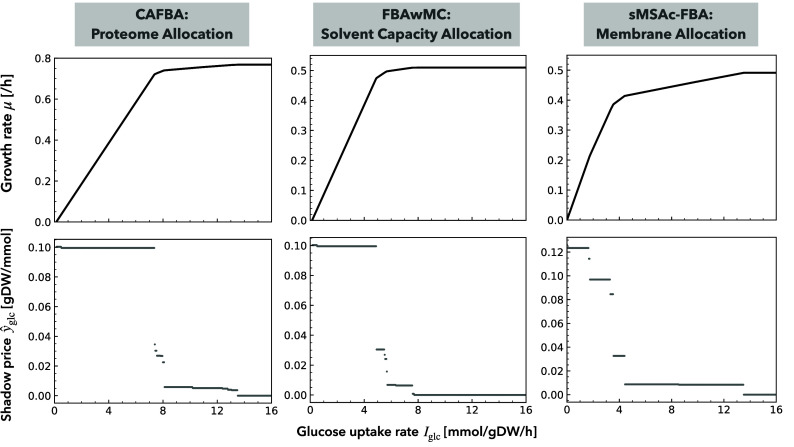
Growth rate μ (*Top*) and shadow price ${\hat{y}}_{\text{glc}}$ of glucose (*Bottom*) as a function of carbon source availability $I_{\text{glc}}$. Numerical calculations of various CBM methods with either constraint on the allocation of proteome [constrained allocation flux balance analysis, CAFBA (27); see also *SI Appendix*, Fig. S1], cytoplasmic volume [FBA with molecular crowding, FBAwMC (30, 31)], or membrane surface area [specific membrane surface area-constrained FBA, sMSAc-FBA (32)] were performed. See *Materials and Methods* for details.

To confirm the above analytical results, we also performed numerical simulations of various CBM methods with genome-scale metabolic networks; they include either constraint on the allocation of proteins (27), cytoplasmic volume (30, 31), or membrane surface area (32) (see *Materials and Methods* for details). The resulting piecewise linear growth kinetics curve $\mu (I_{\text{glc}})$ is indeed monotonically increasing and concave, as its slope ${\hat{y}}_{\text{glc}}\;=\;\partial \mu /\partial I_{\text{glc}}$ monotonically decreases to zero (Fig. 2).

Each locally linear part of the microbial growth kinetics curve represents a distinct phenotype phase, i.e., a qualitatively different metabolic phenotype with a different combination of growth-limiting constraints (39). Due to the limited amount of protein, intracellular volume, or membrane surface area (i.e., a factor in C), carbon metabolism at intermediate $I_{\text{glc}}$ reallocates enzymes to less efficient metabolic reactions in terms of growth yield, which gradually diminishes the growth return from additional glucose intake. In the examples of CBMs in Fig. 2, with small $I_{\text{glc}}$, only carbon source intake is the growth-limiting factor. Finally, the availability of resources other than glucose determines the maximum growth rate.

### Growth Rate Dependence on Multiple Nutrients.

The global constraint principle not only reproduces the known fundamental characteristics (i-ii) of the microbial growth kinetics curve but also leads to a phenomenological theory for the dependence of growth rate on multiple nutrients.

**Fig. 3.**
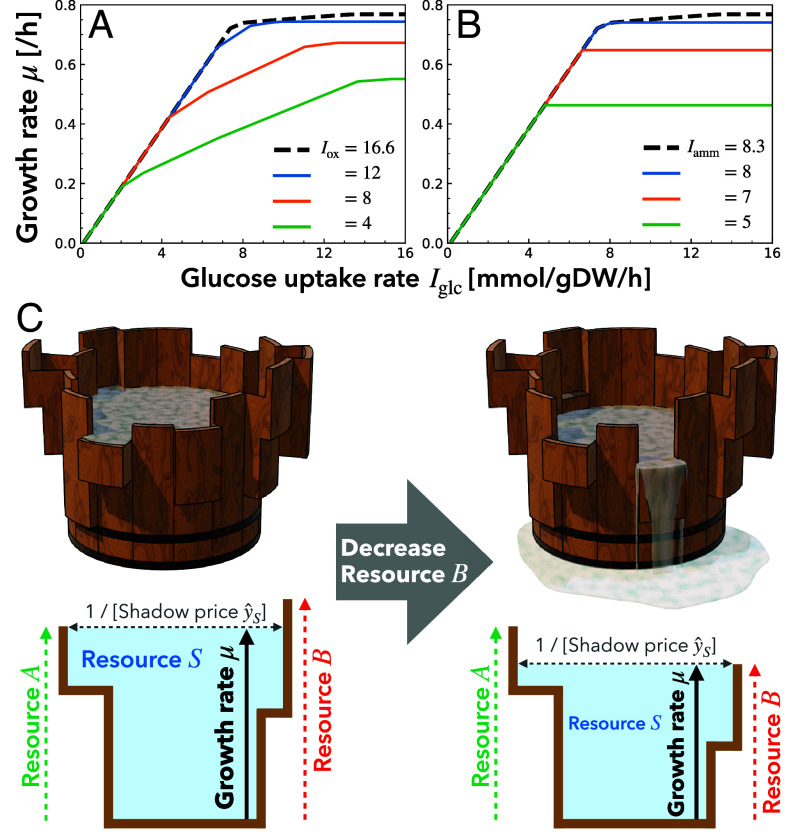
Growth rate μ as a function of carbon source availability $I_{\text{glc}}$ with different maximal influxes of (*A*) oxygen $I_{\text{ox}}$ and (*B*) nitrogen source $I_{\text{amm}}$. The dashed lines correspond to the case with $I_{\text{ox}}\;=\;16.6$ and $I_{\text{amm}}\;=\;8.3$. Numerical calculations of CAFBA (27) were performed using the genome-scale *E. coli* iJO1366 model (46) and the COBRApy package (47) (see *SI Appendix*, Text S3.A for details). (*C*) Schematic with terraced Liebig’s barrels (Fig. 1*C*). (*Left*) The shortest stave corresponds to resource A, which has the lowest availability, and thus it determines the maximum growth rate. (*Right*) Due to a decrease in the availability of resource B, the maximum growth rate is limited by B. It leads to the reallocation of B to metabolic processes and the resulting decrease in the shadow price ${\hat{y}}_{S}\;=\;\partial \mu /\partial I_{S}$ at a lower $I_{S}$.

Since the growth kinetics curve $\mu (I_{S};\;\tilde{I})$ is shaped by the global regulation of intracellular metabolism, it depends not only on the availability of the focal nutrient S but also on that of the other resources, here denoted as $\tilde{I}$. If the availability of a nonfocal resource, i.e., a resource other than S, increases (decreases), the constraint on the allocation of the nonfocal resource is relaxed (tightened); as a result, the growth kinetics curve $\mu (I_{S};\;\tilde{I})$ should shift up (down) in the phase(s) where cell growth is limited by the nonfocal resource (Fig. 3*C*). Conversely, as an extension of classical ideas proposed by Monod (5) and Liebig (3, 16, 20), one can empirically infer the growth-limiting factors under the original environmental conditions by measuring how the shape of the microbial growth kinetics curve responds to environmental manipulations, such as the addition of a nutrient to the medium.

Such responses of the microbial growth kinetics curve are indeed observed numerically (Fig. 3). The decrease in the availability of a nonfocal resource, oxygen $I_{\text{ox}}$ (Fig. 3*A*) or a nitrogen source (ammonium) $I_{\text{amm}}$ (Fig. 3*B*), shifts the growth kinetics curve $\mu (I_{\text{glc}})$ downward in a phase with sufficiently large $I_{\text{glc}}$, while maintaining the same slope ${\hat{y}}_{S}\;=\;\partial \mu /\partial I_{S}$ until the initial downward shift. Then, one can empirically conclude that cell growth in this phase is limited by the nonfocal resource under the original environment. These behaviors, which are not captured by Monod’s law, can be tested experimentally. In fact, a nitrogen-limited chemostat experiment exhibited that a decrease in the nitrogen source availability gradually shifts the growth kinetics curve $\mu (I_{\text{glc}})$ downward, while maintaining the same slope ${\hat{y}}_{\text{glc}}\;=\;\partial \mu /\partial I_{\text{glc}}$ until the initial downward shift in a phase with sufficiently large $I_{\text{glc}}$ (48) (*SI Appendix*, Fig. S2). Such earlier experimental studies yielded qualitatively consistent results, while further experiments using modern techniques, like a carbon- and nitrogen-limited chemostat experiment in ref. 49, will be essential for rigorous validation.

## Discussion

In this study, we have shown that the fundamental characteristics of the microbial growth kinetics curve can be explained as general properties of optimal resource allocation in cellular metabolism: As the availability of a nutrient increases, its metabolism into biomass becomes constrained by the intracellular allocation of other resources, which gradually diminishes the growth return from the additional intake of the nutrient (Fig. 1 *B* and *C*). Note that microbial growth kinetics curves are often naively assumed to be smooth functions in the form of the Monod equation (Eq. **1**); however, apparently multiphasic growth kinetics curves, which are nonsmooth at the boundaries between different phenotype phases, have been observed experimentally (29, 44, 45). The global constraint principle can describe the microbial growth kinetics curve with arbitrary precision by a piecewise linear function, at least if a sufficient number of constraints are considered.

While previous studies have often argued specific (nonnutrient) constraints on the relationship between cellular growth and metabolism (27–31, 36, 50), our approach explores a universality that is not necessarily attributable to specific molecular-biological mechanisms. This approach is in line with the physics of living systems and has been established in previous research on the “microeconomics of metabolism” (25, 36).

The global constraint principle for microbial growth kinetics also provides a theoretical basis for the dependence of growth rate on the availability of multiple nutrient sources, which cannot be captured by the classical arguments about Monod’s bacterial growth model. In particular, we elucidated how the microbial growth kinetics curve $\mu (I_{S})$ responds to manipulating the environmental availability of a nutrient other than the focal nutrient S. Remarkably, this model can be viewed as a generalization of Liebig’s law of the minimum, which states that the growth rate of an organism is determined by the availability of the scarcest resource. Liebig’s law is commonly illustrated by the metaphor of a barrel with uneven staves (51), as the feasible water surface height of the barrel is limited by the shortest stave. This barrel metaphor describes the effect of resource availability on the maximum growth rate but does not account for growth kinetics. In contrast, the global constraint principle, which incorporates the influence of resource allocation on growth kinetics, is also illustrated schematically using a modified Liebig’s barrel with terraced staves (Fig. 1*C*): At the water level where an additional global constraint appears, each stave of the terraced Liebig’s barrel spreads out in a stepwise manner, making further increases in growth rate more difficult.

Future research could explore a unified framework connecting the global constraint principle with growth laws beyond Monod’s and Liebig’s. For instance, as our theory provides a general proof of the concavity of the growth rate in the coutilization of arbitrary nutrient sources, a promising direction would be to expand this approach to derive more quantitative and predictive growth laws for nutrient coutilization (52). Another intriguing possibility is to establish a connection between our framework and population growth laws describing the dependence of growth rate on population size (53, 54). This would require an additional assumption about how nutrient availability scales with population size, but it could offer valuable insights into the dynamics of populations limited by resources.

Finally, we acknowledge certain limitations. This study examines how optimized metabolic regulation universally imposes the monotonic and concave relationship between nutrient availability and growth rates. Therefore, if the optimization assumption fails, for example due to the toxicity of acids and alcohols at high concentrations (55), the monotonicity may break down (15, 55); in addition, metabolic regulation may not be fully optimized at low growth rates (56), which may result in violations of monotonicity or concavity in the low-growth regimes. Conversely, under the global constraint principle, systematic deviations of experimental data from monotonicity or concavity indicate the presence of factors influencing cellular growth beyond the optimal allocation of limited resources.

The present study revisited the classical phenomenological laws in biology, Monod’s law of bacterial growth and Liebig’s law of the minimum, from the perspective of resource allocation in cellular metabolism. We have thereby refined them into a comprehensive theory of the law of diminishing returns in cellular growth. As Liebig’s law was originally formulated to describe the growth of plants, we expect our theory to provide a theoretical basis for the growth of higher organisms as well as microbes in the future.

## Materials and Methods

In this paper, three CBM methods were used to numerically demonstrate our general proof (Figs. 2 and 3). While all of these methods can be formally represented as Eqs. **2**–**4**, we here explain the details of each method. The mass balance due to intracellular reactions (Eqs. **2** and **3**) is essentially the same in all CBM methods, while the nonnutrient resources C and the corresponding constraints (Eq. **4**) differ across methods.

### Constrained Allocation Flux Balance Analysis (CAFBA).

In the method of CAFBA (27), the allocation of the proteins associated with ribosomes, biosynthetic enzymes, carbon transport, and housekeeping reaction processes is introduced as $\phi_{R}$, $\phi_{E}$, $\phi_{C}$, and $\phi_{Q}$, respectively. The sum of these proteome fractions is normalized such that $\phi_{R}+\phi_{E}+\phi_{C}+\phi_{Q}=1$.

The proteome fractions, except for the housekeeping process fraction $\phi_{Q}$, vary with environmental conditions and physiological states as follows: The ribosome sector $\phi_{R}$ increases proportionally to biomass synthesis rate $v_{\text{gr}}$ as $\Delta \phi_{R}=\;C_{\phi ,\text{gr}}v_{\text{gr}}$; the biosynthetic enzyme sector $\phi_{E}$ changes proportionally to the reaction flux $v_{i}$ such that $\Delta \phi_{E}=\;\sum_{i}C_{\phi ,i}v_{i}$; the carbon transport sector $\phi_{C}$ changes proportionally to the carbon intake flux as $\Delta \phi_{C}=\;C_{\phi ,C}v_{C}$.

Accordingly, the constraint for partitioning the flexible proteome fractions in CAFBA is represented as

$$\begin{array}{c} \Delta \phi_{R}+\Delta \phi_{E}+\Delta \phi_{C}=\sum_{i\in R}C_{\phi ,i}v_{i}\leq I_{\phi }. \end{array}$$

$I_{\phi }$ is set to 0.4 and the parameter values of $C_{\phi ,i}$ are calibrated according to the protocol in ref. 57.

### Flux Balance Analysis with Molecular Crowding (FBAwMC).

The method of FBAwMC (30, 31) incorporates the limited solvent capacity for the allocation of metabolic enzymes. Since the enzyme molecules have a finite molar volume $V_{i}$, only a finite number of them can fit in a given cell volume V. That is, if $n_{i}$ is the number of moles of enzyme i, then $\sum_{i}V_{i}n_{i}\leq V$.

Additionally, it is also assumed that an enzyme concentration $E_{i}:=\;n_{i}/M$ (moles per unit mass; M is cell mass) results in a flux $v_{i}=\;b_{i}E_{i}$ for each reaction i, where the parameter $b_{i}$ is determined by the reaction mechanism, kinetic parameters, and metabolite concentrations. Then, the enzyme concentration constraint due to molecular crowding is reflected as the following metabolic flux constraint:

$$\begin{array}{c} \sum_{i\in R}C_{V,i}v_{i}\leq 1=:I_{V},\;C_{V,i}:=\frac{MV_{i}}{Vb_{i}}. \end{array}$$

According to ref. 30, $C_{V,i}$’s are given as random values from the gamma distribution P(a) with the average ⟨a⟩≃0.0031 [h·g/mmol] and the shape parameter β=3; note that for β≫1, the resultant distribution is almost concentrated around a=⟨a⟩. In the numerical simulation of Fig. 2, the stoichiometry matrix S is based on the genome-scale *E. coli* iJO1366 model (46).

### Specific Membrane Surface Area-constrained Flux Balance Analysis (sMSAc-FBA).

The method of sMSAc-FBA (32) incorporates the limited capacity of specific membrane surface area (sMSA) to host proteins. Given the finite surface area-to-volume ratio (28, 32), denoted as $I_{\text{MRE}}$, the corresponding constraint is represented as

$$\begin{array}{c} \sum_{i\in R}C_{\text{MRE},i}v_{i}\leq I_{\text{MRE}}, \end{array}$$

where “membrane real estate” coefficient $C_{\text{MRE},i}$ [h · nm^2^/mmol] denotes the membrane area used by the active enzymes for reaction i. According to ref. 32, the parameter values of $C_{\text{MRE},i}$ were chosen based on the intracellular stoichiometry derived from the *E. coli* core model, and $I_{\text{MRE}}$ is specified as 2.8 [nm^2^/gDW].

## Supplementary Material

Appendix 01 (PDF)

## Data Availability

There are no data underlying this work.

## Footnotes

^*^If the substrate intake function $I_{S}\left(\left[S\right]\right)$ is nonconcave—e.g., in the case of n-mer transport mechanisms modeled as the Hill equation $I_{S}\;\propto \;{\left[S\right]}^{n}/\left(K_{\text{S}}\;+\;{\left[S\right]}^{n}\right)$—then $\mu \left(\left[S\right]\right)\;=\;\mu \left(I_{S};\;\tilde{I}\right)\;\circ \;I_{S}\left(\left[S\right]\right)$ may lose concavity, especially in low-substrate regimes. However, such nonconcave transport of growth-limiting substrates is likely rare in most biological contexts.
