# Supplementary material for: Global constraint principle for microbial growth laws
Source: Proc Natl Acad Sci U S A. 2025 Oct 3;122(40):e2515031122. doi: 10.1073/pnas.2515031122 (PMC12519149; doi:10.1073/pnas.2515031122)
Supplement: Supplementary file 1 — Appendix 01 (PDF) [file pnas.2515031122.sapp.pdf]

Supporting Information for

Global Constraint Principle for Microbial Growth Laws

Jumpei F. Yamagishi and Tetsuhiro S. Hatakeyama

Jumpei F. Yamagishi.
E-mail: jumpei.yamagishi@riken.jp
Tetsuhiro S. Hatakeyama.
E-mail: hatakeyama@elsi.jp

This PDF file includes:

Supporting text
Figs. S1 to S3
SI References

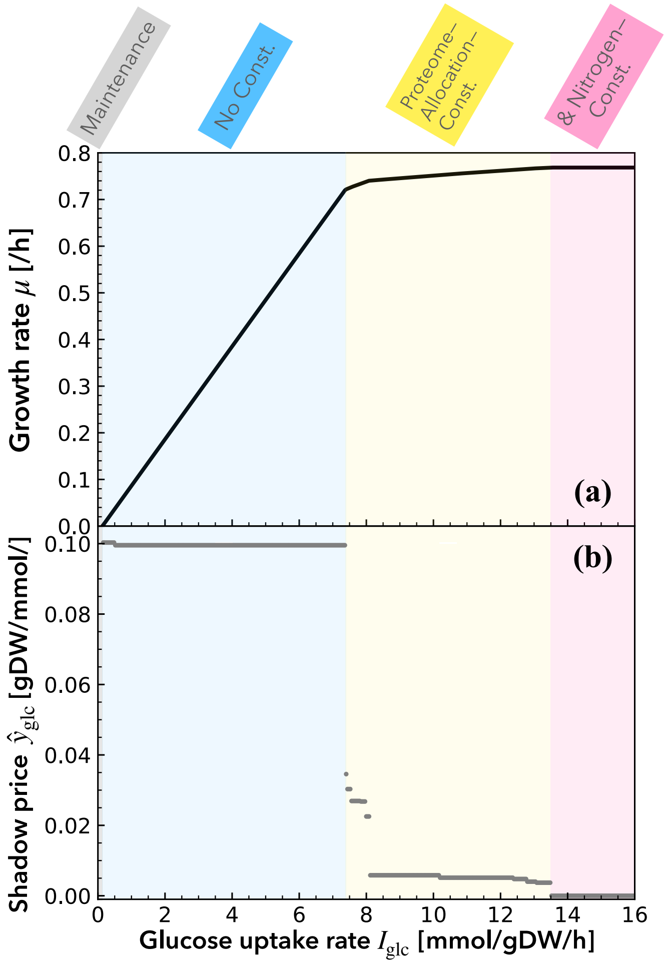

Fig. S1. (a) Growth rate μ and (b) shadow price $\hat{y}_{\mathrm{glc}}$ of glucose as a function of carbon source availability I_{glc} with $I_{\mathrm{amm}} = 8.3$. Numerical calculations using a CBM method with proteome allocation, constrained allocation flux balance analysis (CAFBA) (1), were performed using the genome-scale *E. coli* iJO1366 model (2) and the COBRApy package (3). See also Materials and Methods and Fig. 2 in the main text.

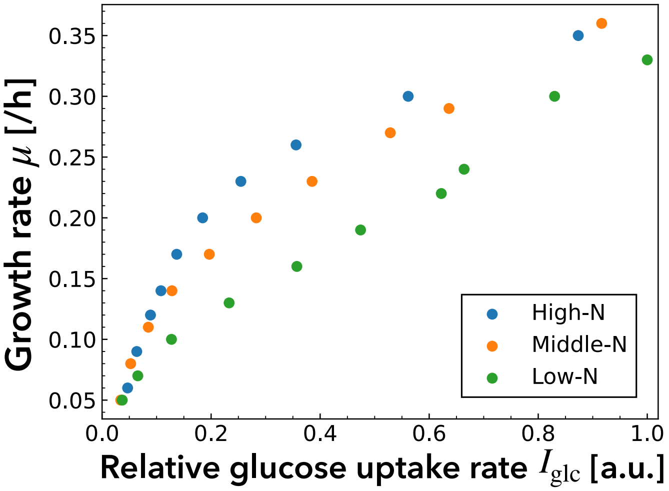

Fig. S2. Dependence of growth rates of *S. cerevisiae* on the relative glucose uptake rate with different nitrogen source availability. Using a nitrogen-limited chemostat, this experiment manipulates the availability of the nitrogen source, $(\mathrm{NH_4})_2\mathrm{SO_4}$, to achieve high-, middle-, or low-nitrogen concentration conditions (see Larsson et al. (4) for more details); however, we note that the C/N ratios of the supplied medium in the middle- and low-N conditions were not fixed across different dilution rates of the chemostat in the experimental protocol. The values in this plot are calculated from Fig. 2 of Larsson et al. (4); since the authors did not report cellular dry mass even though they measured it, and only growth yield and chemostat dilution rate were provided, we could only calculate the relative glucose uptake rates, which are proportional to the growth rates divided by the growth yields, not the absolute values. Finally, we note that Figs. 3a and 3b in the main text show numerical simulations based on an *E. coli* metabolic model, whereas this figure uses yeast experimental data. While this is not a strictly consistent comparison, multiphasic growth and changes in growth-curve slope are general features.

Supporting Information Text

S1. Derivation of the dual problem (Eq. 5) in the main text

To derive the dual problem, let us introduce matrices

$$\begin{aligned} \mathsf{N}^{\mathrm{int}} &:= -\mathsf{S}^{\mathcal{M}\setminus\mathcal{E}} = \{-\mathsf{S}_{ai}\}_{a\in\mathcal{M}\setminus\mathcal{E}, i\in\mathcal{R}}, \\ \mathsf{N}^{\mathrm{ex}} &:= \begin{pmatrix} -\mathsf{S}^{\mathcal{E}} \\ \mathsf{C} \end{pmatrix}, \end{aligned}$$

with $\mathsf{S}^{\mathcal{E}} := \{\mathsf{S}_{ai}\}_{a\in\mathcal{E}, i\in\mathcal{R}}$, and rewrite the linear programming (LP) problem (2-4) as

$$\underset{\mathbf{v}}{\text{maximize}}\ v_{\mathrm{gr}}\ \ \text{s.t.}\ \ \mathsf{N}^{\mathrm{int}}\mathbf{v} = \mathbf{0},\ \ \mathsf{N}^{\mathrm{ex}}\mathbf{v} \le \mathbf{I},\ \ \mathbf{v} \ge \mathbf{0}.$$

Then, the dual problem to this primal LP program is formally derived as

$$\min_{\mathbf{y}\in\mathbb{R}^{\mathcal{M}\cup\mathcal{C}}} \begin{pmatrix} \mathbf{0} \\ \mathbf{I} \end{pmatrix} \cdot \mathbf{y} \quad \text{s.t.} \quad \begin{pmatrix} \mathsf{N}^{\mathrm{int}\top} & \mathsf{N}^{\mathrm{ex}\top} \\ \mathbf{0} & \mathsf{I} \end{pmatrix} \mathbf{y} \ge \mathbf{1}_{\mathrm{gr}},$$

where I is the $(\mathcal{E}\cup\mathcal{C})\times(\mathcal{E}\cup\mathcal{C})$ identity matrix and $\mathbf{1}_{\mathrm{gr}} \in \{0,1\}^{\mathcal{R}\cup\mathcal{E}\cup\mathcal{C}}$ denotes a vector where the gr-th element is unity and all other elements are zero (5–7). This can be rewritten as

$$\begin{aligned} &\underset{\mathbf{y}\in\mathbb{R}^{\mathcal{M}\cup\mathcal{C}}}{\text{minimize}} \sum_{m\in\mathcal{E}\cup\mathcal{C}} I_m y_m \\ &\text{s.t.} \quad -\sum_{m\in\mathcal{M}} \mathsf{S}_{mi} y_m + \sum_{m\in\mathcal{C}} \mathsf{C}_{mi} y_m \ge 0 \quad (i \in \mathcal{R}\setminus\{\mathrm{gr}\}), \\ &\qquad -\sum_{m\in\mathcal{M}} \mathsf{S}_{m,\mathrm{gr}} y_m + \sum_{a\in\mathcal{C}} \mathsf{C}_{m,\mathrm{gr}} y_m \ge 1, \\ &\qquad y_a \ge 0 \ \ (a \in \mathcal{E}\cup\mathcal{C}). \end{aligned}$$

It is equivalent to the dual problem (Eq. 5) in the main text.

A. A simple example of the duality. As a concrete example, we present here an analytically-solvable metabolic model (Fig. S3). It is a simple metabolic model where multiple pathways produce a common metabolite from a single nutrient: e.g., the co-utilization of respiration and fermentation (8) or that of Embden–Meyerhoff–Parnass (EMP) and Entner–Doudoroff (ED) glycolytic pathways (9).

This model consists of 2 metabolites, $\mathcal{M} = \{S, P\}$, and 1 constraint $\mathcal{C} = \{\rho\}$, as well as 3 reactions, $\mathcal{R} = \{A, B, \mathrm{gr}\}$. Note that ρ can be any kind of limited (non-nutrient) resource, such as the intracellular volume or solvent capacity (10, 11), the total amount of proteins (12), and the total area of membrane surfaces (13). We assume that S is the only exchangeable metabolite, i.e., $\mathcal{E} = \{S\}$. Reactions A and B both produce P from S by using non-nutrient resource ρ, and the growth reaction gr requires both P and ρ (Fig. S3). The stoichiometry matrix, $\mathsf{S} \in \mathbb{R}^{\mathcal{M}\times\mathcal{R}}$, and the resource constraint matrix, $\mathsf{C} \in \mathbb{R}^{\mathcal{C}\times\mathcal{R}}$, are then given as

$$\begin{aligned} \mathsf{S} &= \begin{pmatrix} -1 & -1 & 0 \\ s_{PA} & s_{PB} & -s_{P\mathrm{gr}} \end{pmatrix}, \\ \mathsf{C} &= \begin{pmatrix} \mathsf{C}_{\rho A} & \mathsf{C}_{\rho B} & \mathsf{C}_{\rho \mathrm{gr}} \end{pmatrix}. \end{aligned}$$

As discussed in ref. (8), a trade-off between reactions A and B leads to the co-utilization of these pathways and switching between them. Therefore, we here assume a trade-off $s_{PA} > s_{PB}$ and $\mathsf{C}_{\rho A} > \mathsf{C}_{\rho B}$: i.e., reaction A is more efficient in producing P but requires more amount of constraint ρ than the alternative reaction B. When this trade-off matters, the optimal flux $\hat{v}_A$ of reaction A is suppressed by increasing maximal nutrient influx I_S.

With matrices S and C, the LP problem for CBM is represented as

$$\begin{aligned} &\underset{\mathbf{v}\ge\mathbf{0}}{\text{maximize}}\ v_{\mathrm{gr}} \quad \text{s.t.} \\ &\qquad -v_A - v_B + I_S \ge 0, \\ &\qquad s_{PA} v_A + s_{PB} v_B - s_{P\mathrm{gr}} v_{\mathrm{gr}} = 0, \\ &\qquad \mathsf{C}_{\rho A} v_A + \mathsf{C}_{\rho B} v_B + \mathsf{C}_{\rho\mathrm{gr}} v_{\mathrm{gr}} \le I_\rho. \end{aligned} \tag{S1}$$

Its solution, or the optimal fluxes $\hat{\mathbf{v}}$, is calculated as

$$(\hat{v}_A, \hat{v}_B) = \begin{cases} (I_S, 0) & \text{if } A_0 > I_S \ge 0 \\ \left(\frac{B_0 - I_S}{B_0 - A_0} A_0,\ \frac{I_S - A_0}{B_0 - A_0} B_0\right) & \text{if } B_0 > I_S \ge A_0 \\ (0, B_0) & \text{if } I_S \ge B_0 \end{cases},$$

where $A_0 := I_\rho/(s_{PA} + \mathsf{C}_{\rho A})$ and $B_0 := I_\rho/(s_{PB} + \mathsf{C}_{\rho B})$. The dependence of the growth rate on I_S is then calculated as

$$\mu(I_S; I_\rho) = \begin{cases} s_{PA} I_S & \text{if } A_0 > I_S \geq 0 \\ s_{PA} \frac{B_0 - I_S}{B_0 - A_0} A_0 + s_{PB} \frac{I_S - A_0}{B_0 - A_0} B_0 & \text{if } B_0 > I_S \geq A_0 \\ s_{PB} B_0 & \text{if } I_S \geq B_0 \end{cases} . \quad \text{[S2]}$$

It indeed satisfies monotonicity and concavity (see also Fig. S3).

The dual problem to the primal LP problem (Eq. S1) is

$$\begin{aligned} \underset{\mathbf{y} \geq \mathbf{0}}{\text{minimize}} \quad & I_S y_S + I_\rho y_\rho \\ \text{s.t.} \quad & y_S - s_{PA} y_P \geq 0, \\ & y_S - s_{PB} y_P + \mathsf{C}_{\rho B} y_\rho \geq 0, \\ & s_{P\mathrm{gr}} y_P + \mathsf{C}_{\rho,\mathrm{gr}} y_\rho \geq 1. \end{aligned} \quad \text{[S3]}$$

The dual problem is formal in nature and, unlike the original problem, is difficult to interpret biologically. However, an intuitive (economic) interpretation of this minimization problem is to find the smallest "selling price that avoids incurring a loss," which is the solution of the dual problem or shadow price $\hat{\mathbf{y}}$.

The shadow price $\hat{y}_S$ of nutrient S is calculated as

$$\hat{y}_S(I_S; I_\rho) = \begin{cases} s_{PA} & \text{if } I_S \leq A_0 \\ \frac{s_{PB} B_0 - s_{PA} A_0}{B_0 - A_0} & \text{if } B_0 \geq I_S \geq A_0 \\ 0 & \text{if } I_S \geq B_0 \end{cases} .$$

It is indeed monotonically decreasing.

Note that the dual LP problem (Eq. S3) is different from minimizing the nutrient influx I_S to achieve a given growth rate μ, which is often considered in constraint-based modeling (CBM):

$$\begin{aligned} \underset{I_S, v_A, v_B \geq 0}{\text{minimize}} \quad I_S \quad \text{s.t.} \quad & -v_A - v_B + I_S \geq 0, \\ & s_{PA} v_A + s_{PB} v_B - s_{P\mathrm{gr}} v_{\mathrm{gr}} = 0, \\ & \mathsf{C}_{\rho A} v_A + \mathsf{C}_{\rho B} v_B + \mathsf{C}_{\rho\mathrm{gr}} v_{\mathrm{gr}} \leq I_\rho, \\ & v_{\mathrm{gr}} \geq \mu, \end{aligned}$$

where the variables are nutrient influx I_S and reaction fluxes v_A and v_B, while growth rate μ and I_ρ are given as parameters.

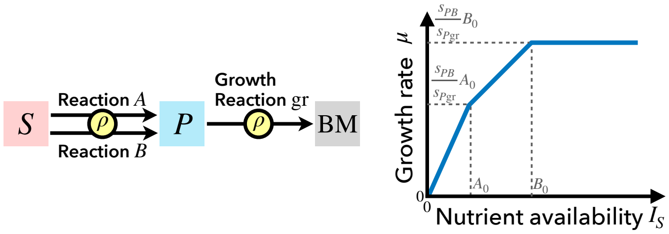

Fig. S3. A simple example of metabolic systems.

B. Representation of growth kinetics curves $\mu(I_S)$ using the shadow price. From the strong duality theorem, $\hat{y}_m = \partial\mu/\partial I_m$ holds. Accordingly, the growth kinetics curve can be formally written as

$$\mu(I_S; \tilde{\mathbf{I}}) = \int_{I_{S,\min}}^{I_S} \hat{y}_S(I_S; \tilde{\mathbf{I}}) \mathrm{d}I_S,$$

which emphasizes that the curve is determined by the cumulative marginal growth return from nutrient uptake. This $\mu(I_S)$ generally represents a multiphasic, piecewise linear function. It can be also represented as follows:

$$\mu(I_S; \tilde{\mathbf{I}}) = \begin{cases} \hat{y}_S(I_{S,\min})(I_S - I_{S,\min}) & \text{if } I_{S,1} > I_S \geq I_{S,\min} \\ \hat{y}_S(I_{S,1})(I_S - I_{S,1}) + \hat{y}_S(I_{S,\min})(I_{S,1} - I_{S,\min}) & \text{if } I_{S,2} > I_S \geq I_{S,1} \\ \hat{y}_S(I_{S,2})(I_S - I_{S,2}) + \hat{y}_S(I_{S,1})(I_{S,2} - I_{S,1}) + \hat{y}_S(I_{S,\min})(I_{S,1} - I_{S,\min}) & \text{if } I_{S,3} > I_S \geq I_{S,2} \\ \cdots & \\ \hat{y}_S(I_{S,n})(I_S - I_{S,n}) + \sum_{k=0}^{n-1} \hat{y}_S(I_{S,k})(I_{S,k+1} - I_{S,k}) & \text{if } I_{S,n+1} > I_S \geq I_{S,n} \\ \cdots & \\ \sum_{k=0}^{N-1} \hat{y}_S(I_{S,k})(I_{S,k+1} - I_{S,k}) & \text{if } I_S \geq I_{S,N} \end{cases} , \quad \text{[S4]}$$

where $I_{S,0} := I_{S,\min}$. Notably, $I_{S,i}$ $(i=1,\cdots,N)$ depends on the details of CBM problems (see also Eq. S2 for a simple example).

S2. Proof of the concavity of optimal objective function

The concavity of optimal objective function $\hat{v}_{\rm gr}(\mathbf{I})$ as a multivariable function of $\mathbf{I}$ can be analytically proven as follows.

From the LP duality theorem (6) and the dual problem (Eq. 5) in the main text,

$$\hat{v}_{\rm gr}(\mathbf{I}) = \min_{\mathbf{y}\in\mathbb{R}^{\mathcal{M}\cup\mathcal{C}}} \{\mathbf{I}\cdot\mathbf{y}_{\mathcal{E}\cup\mathcal{C}} | \begin{pmatrix} -\mathsf{S}^\top & \mathsf{C}^\top \end{pmatrix} \mathbf{y} \geq \mathbf{1}_{\rm gr}, \mathbf{y}_{\mathcal{E}\cup\mathcal{C}} \geq \mathbf{0}\},$$

where $\mathbf{y}_{\mathcal{E}\cup\mathcal{C}}$ is defined as $\mathbf{y}_{\mathcal{E}\cup\mathcal{C}} := \{y_a\}_{a\in\mathcal{E}\cup\mathcal{C}}$ and $\mathbf{1}_{\rm gr} \in \{0,1\}^{\mathcal{R}}$ denotes a vector where the gr-th element is unity and all other elements are zero. In contrast to the primal problem (2-4) in the main text, it is useful that the feasible solution space of the dual problem (Eq. 5) in the main text does not change when $\mathbf{I}$ is varied.

Thus, for arbitrary $0 < t < 1$ and $\mathbf{I}_1, \mathbf{I}_2$,

$$\begin{aligned}
&\hat{v}_{\rm gr}(t\mathbf{I}_1 + (1-t)\mathbf{I}_2) \\
&= \min_{\mathbf{y}} \{(t\mathbf{I}_1 + (1-t)\mathbf{I}_2)\cdot\mathbf{y}_{\mathcal{E}\cup\mathcal{C}} | \begin{pmatrix} -\mathsf{S}^\top & \mathsf{C}^\top \end{pmatrix} \mathbf{y} \geq \mathbf{1}_{\rm gr}, \mathbf{y}_{\mathcal{E}\cup\mathcal{C}} \geq \mathbf{0}\} \\
&\geq \min_{\mathbf{y}} \{t\mathbf{I}_1\cdot\mathbf{y}_{\mathcal{E}\cup\mathcal{C}} | \begin{pmatrix} -\mathsf{S}^\top & \mathsf{C}^\top \end{pmatrix} \mathbf{y} \geq \mathbf{1}_{\rm gr}, \mathbf{y}_{\mathcal{E}\cup\mathcal{C}} \geq \mathbf{0}\} \\
&\quad + \min_{\mathbf{y}} \{(1-t)\mathbf{I}_2\cdot\mathbf{y}_{\mathcal{E}\cup\mathcal{C}} | \begin{pmatrix} -\mathsf{S}^\top & \mathsf{C}^\top \end{pmatrix} \mathbf{y} \geq \mathbf{1}_{\rm gr}, \mathbf{y}_{\mathcal{E}\cup\mathcal{C}} \geq \mathbf{0}\} \\
&= t\hat{v}_{\rm gr}(\mathbf{I}_1) + (1-t)\hat{v}_{\rm gr}(\mathbf{I}_2).
\end{aligned}$$

This is the definition of the concavity.

A. Proof of the concavity in nonlinear CBM. The above proof of the concavity in linear programs using the duality theorem can be extended to nonlinear convex programs.

In general, by adding M convex constraints to the LP program (2-4) in CBM, convex optimization problems in CBM can be formulated as:

$$\begin{aligned}
\underset{\mathbf{v},\mathbf{x}\geq\mathbf{0}}{\text{maximize}}\ v_{\rm gr} \quad \text{s.t.} \quad & \mathsf{N}^{\rm int}\mathbf{v} = \mathbf{0}, \\
& \mathsf{N}^{\rm ex}\mathbf{v} \leq \mathbf{I}, \\
& g_\alpha(\mathbf{v},\mathbf{x}) \leq 0 \ \ (\alpha = 1,\cdots,M).
\end{aligned} \tag{S5}$$

The variables $\mathbf{x}$ typically represent metabolite concentrations, but can include other variables; here, $g_\alpha(\mathbf{v},\mathbf{x})$ is assumed to be a convex function and represents, for example, constraints in the relationship between concentrations $\mathbf{x}$ and fluxes $\mathbf{v}$.

An example of a nonlinear convex optimization problem in CBM is Conic FBA (14), which incorporates the relationship between reaction fluxes and metabolite concentrations as constraints and models cellular metabolism and growth as a second-order cone program. In this method, the Michaelis–Menten kinetics is represented as a convex function $g_\alpha(v_\alpha,\mathbf{x})$ (see refs. (14, 15) for more details).

To prove the concavity of $\hat{v}_{\rm gr}(\mathbf{I})$, it is convenient to consider the Lagrange dual problem of the convex program (Eq. S5):

$$\underset{\mathbf{y}}{\text{minimize}} \sum_{a\in\mathcal{E}\cup\mathcal{C}} I_a y_a + \max_{\mathbf{v},\mathbf{x}\geq\mathbf{0}} \sum_\alpha g_\alpha(\mathbf{v},\mathbf{x}) y_\alpha \tag{S6}$$

$$\text{s.t.} \begin{pmatrix} \mathsf{N}^{\rm int\top} & \mathsf{N}^{\rm ex\top} \\ \mathbf{0} & \mathsf{I} \end{pmatrix} \mathbf{y} \geq \mathbf{1}_{\rm gr}, \ \ y_\alpha \geq 0 \ \ (\alpha = 1,\cdots,M). \tag{S7}$$

As with the LP dual problem, note that the parameter $\mathbf{I}$ appears only as a coefficient in the objective function and does not affect the feasible solution. From the strong duality theorem (16), for arbitrary $0 < t < 1$ and $\mathbf{I}_1, \mathbf{I}_2$,

$$\begin{aligned}
&\hat{v}_{\rm gr}(t\mathbf{I}_1 + (1-t)\mathbf{I}_2) \\
&= \min_{\mathbf{y}} \{(t\mathbf{I}_1 + (1-t)\mathbf{I}_2)\cdot\mathbf{y}_{\mathcal{E}\cup\mathcal{C}} + \sum_\alpha g_\alpha(\hat{\mathbf{v}},\hat{\mathbf{x}}) y_\alpha | \text{Eq. (S7)}\} \\
&\geq \min_{\mathbf{y}} \{t\mathbf{I}_1\cdot\mathbf{y}_{\mathcal{E}\cup\mathcal{C}} + t\sum_\alpha g_\alpha(\hat{\mathbf{v}},\hat{\mathbf{x}}) y_\alpha | \text{Eq. (S7)}\} \\
&\quad + \min_{\mathbf{y}} \{(1-t)\mathbf{I}_2\cdot\mathbf{y}_{\mathcal{E}\cup\mathcal{C}} + (1-t)\sum_\alpha g_\alpha(\hat{\mathbf{v}},\hat{\mathbf{x}}) y_\alpha | \text{Eq. (S7)}\} \\
&= t\hat{v}_{\rm gr}(\mathbf{I}_1) + (1-t)\hat{v}_{\rm gr}(\mathbf{I}_2).
\end{aligned}$$

This is the definition of the concavity.

The monotonic increase in the growth kinetics curve $\mu(I_S;\tilde{\mathbf{I}})$ is also evident.

References

1. M Mori, T Hwa, OC Martin, A De Martino, E Marinari, Constrained allocation flux balance analysis. *PLoS Comput. Biol.* **12**, e1004913 (2016).
2. JD Orth, et al., A comprehensive genome-scale reconstruction of *Escherichia coli* metabolism—2011. *Mol. Syst. Biol.* **7**, 535 (2011).
3. A Ebrahim, JA Lerman, BØ Palsson, DR Hyduke, Cobrapy: Constraints-based reconstruction and analysis for python. *BMC Syst Biol* **7** (2013).
4. C Larsson, U von Stockar, I Marison, L Gustafsson, Growth and metabolism of saccharomyces cerevisiae in chemostat cultures under carbon-, nitrogen-, or carbon-and nitrogen-limiting conditions. *J. Bacteriol.* **175**, 4809–4816 (1993).
5. PB Warren, JL Jones, Duality, thermodynamics, and the linear programming problem in constraint-based models of metabolism. *Phys. Rev. Lett.* **99**, 108101 (2007).
6. RJ Vanderbei, *Linear Programming: Foundations and Extensions.* Springer, (2008).
7. E Reznik, P Mehta, D Segrè, Flux imbalance analysis and the sensitivity of cellular growth to changes in metabolite pools. *PLoS Comput. Biol.* **9**, e1003195 (2013).
8. JF Yamagishi, TS Hatakeyama, Microeconomics of metabolism: the Warburg effect as Giffen behaviour. *Bull. Math. Biol.* **83**, 120 (2021).
9. A Flamholz, E Noor, A Bar-Even, W Liebermeister, R Milo, Glycolytic strategy as a tradeoff between energy yield and protein cost. *Proc. Natl. Acad. Sci.* **110**, 10039–10044 (2013).
10. A Vazquez, J Liu, Y Zhou, ZN Oltvai, Catabolic efficiency of aerobic glycolysis: the Warburg effect revisited. *BMC Syst. Biol.* **4**, 1–9 (2010).
11. A Vazquez, *Overflow Metabolism: From Yeast to Marathon Runners.* Academic Press, London, (2017).
12. M Basan, et al., Overflow metabolism in escherichia coli results from efficient proteome allocation. *Nature* **528**, 99–104 (2015).
13. M Szenk, KA Dill, AMR de Graff, Why do fast-growing bacteria enter overflow metabolism? testing the membrane real estate hypothesis. *Cell Syst.* **5**, 95–104 (2017).
14. JA Taylor, A Rapaport, D Dochain, Convex representation of metabolic networks with Michaelis–Menten kinetics. *Bull. Math. Biol.* **86**, 65 (2024).
15. JA Taylor, A Rapaport, Second-order cone optimization of the gradostat. *Comput. & Chem. Eng.* **151**, 107347 (2021).
16. DG Luenberger, Y Ye, *Linear and Nonlinear Programming.* Springer, (2008).
